# Supplementary material for: Pretreatment Serum Levels of IL-1 Receptor Antagonist and IL-4 Are Predictors of Overall Survival in Multiple Myeloma Patients Treated with Bortezomib
Source: J Clin Med. 2021 Dec 26;11(1):112. doi: 10.3390/jcm11010112 (PMC8745099; doi:10.3390/jcm11010112)
Supplement: Supplementary file 1 [file jcm-11-00112-s001.zip › jcm-1503661-supplementary.pdf]

Table S1. Cytokine profile of three identified clusters of multiple myeloma patients. Data are presented as mean values and SD or median and interquartile range (IQR), depending on the variable distribution. P-values from the global test (ANOVA/Kruskal-Wallis test) are reported with post hoc comparisons (Tukey's test/Duun's test) if the p-value of the global test is significant.

**Cytokine**	**Cluster 1**		**Cluster 2**		**Cluster 3**		**ANOVA *p***	**Cluster 1 vs 2 *p***	**Cluster 1 vs 3 *p***	**Cluster 2 vs 3 *p***
	**Mean**	**SD**	**Mean**	**SD**	**Mean**	**SD**				
IL-13	2.97	2.06	2.44	0.93	2.40	2.43	0.6127			
IL-17	23.64	5.76	26.04	10.06	16.44	6.54	0.0002	0.6367	0.0017	0.0014
PDGF-BB	3830.97	1305.15	2790.87	1697.88	2774.65	1064.63	0.0116	0.1019	0.0132	0.9995
**Cytokine**	**Cluster 1**		**Cluster 2**		**Cluster 3**		**Kruskal-Wallis test *p***	**Cluster 1 vs 2 *p***	**Cluster 1 vs 3 *p***	**Cluster 2 vs 3 *p***
	**Median**	**IQR**	**Median**	**IQR**	**Median**	**IQR**				
IL-1β	0.92	0.70<br>1.28	0.95	0.77<br>1.18	0.92	0.49<br>1.54	0.9509			
IL-1ra	86.81	64.82<br>114.14	94.71	86.82<br>213.80	107.25	86.90<br>219.94	0.0717			
IL-2	6.13	5.47<br>7.61	7.31	5.29<br>9.29	4.53	3.03<br>5.08	0.0008	1.0000	0.0068	0.0045
IL-4	7.42	5.85<br>9.44	7.81	5.65<br>8.52	3.41	2.74<br>5.54	0.0000	1.0000	0.0000	0.0024
IL-5	21.91	16.81<br>25.85	28.46	16.12<br>29.41	18.13	12.21<br>29.93	0.4486			
IL-6	2.81	1.25<br>4.30	5.57	2.92<br>6.47	2.96	1.61<br>4.75	0.1287			
IL-7	23.01	19.55<br>27.82	25.84	20.54<br>30.93	14.06	11.28<br>23.04	0.0004	1.0000	0.0026	0.0054
IL-8	8.56	6.45<br>10.60	11.83	9.05<br>23.20	10.29	5.43<br>16.79	0.2327			
IL-9	519.49	476.02<br>570.64	302.43	123.32<br>462.24	457.36	372.13<br>490.54	0.0001	0.0002	0.0023	0.3957
IL-10	10.87	9.36<br>12.08	10.71	7.87<br>11.78	8.84	7.86<br>12.04	0.4269			
IL-12 (p70)	1.26	1.26<br>1.61	-	-	2.65	2.65<br>4.50	1.000			
IL-15	50.04	40.32<br>58.80	71.63	57.22<br>93.77	60.92	40.41<br>93.83	0.1763			
Eotaxin	98.84	80.49<br>139.02	103.01	92.03<br>122.48	49.66	39.11<br>75.40	0.0000	1.0000	0.0001	0.0081
FGF basic	39.51	35.85<br>40.98	42.30	38.80<br>47.32	32.36	28.09<br>39.59	0.0007	0.5336	0.0194	0.0016
G-CSF	385.69	298.48<br>479.70	451.49	197.29<br>677.90	326.01	268.04<br>523.34	0.6091			
GM-CSF	2.60	1.98<br>3.93	2.15	2.01<br>2.31	3.24	2.41<br>4.26	0.1279			
IFN-γ	3.41	2.67<br>4.30	7.94	7.35<br>8.36	4.66	2.67<br>8.27	0.0564			
IP-10	841.10	570.90<br>1018.39	1231.90	635.18<br>1513.88	1373.75	969.08<br>2395.38	0.0034	0.3437	0.0023	1.0000
MCP-1 (MCAF)	27.46	21.05<br>40.55	24.00	18.38<br>28.14	20.01	17.04<br>30.83	0.1231			
MIP-1α	1.98	1.33<br>2.73	2.88	2.265<br>7.43	3.25	1.86<br>4.79	0.0058	0.0135	0.0357	0.9831
MIP-1β	140.95	127.75<br>150.37	92.10	63.20<br>118.96	125.82	109.31<br>135.94	0.0001	0.0001	0.0121	0.1388

RANTES	12785.10	10461.28<br>14692.82	9726.17	6770.13<br>11516.71	10534.02	8542.05<br>11858.24	0.0020	0.0079	0.0109	1.0000
TNF-α	23.21	21.67<br>30.83	31.24	23.90<br>35.41	21.37	17.23<br>28.94	0.0292	0.5292	0.3665	0.0307
VEGF	101.29	74.28<br>263.55	147.09	103.48<br>157.42	103.08	78.39<br>207.15	0.6090			

Table S2. Comparison of clinical variables between identified three clusters of multiple myeloma patients.

Variable	Cluster 1		Cluster 2		Cluster 3		*p*
	N	%	N	%	N	%	
ISS 3	7	30.4	8	80.0	13	52.0	0.0287*
HB < 10 g/dL at diagnosis	8	36.4	4	50.0	8	29.6	0.5627
Calcium > 2.75 mmol/l at diagnosis	4	18.2	1	12.5	7	25.9	0.6548
Creatinine > 2 mg/dL at diagnosis	2	9.1	2	25.0	6	22.2	0.4060
Bone disease	11	50.0	6	66.7	18	66.7	0.4527
Age >70	6	26.1	5	50.0	6	22.2	0.2386
Response to treatment: CR	7	30.4	5	50.0	11	40.7	0.5355
Response to treatment: ≥VGPR	13	56.5	6	60.0	17	63.0	0.8982

*Cluster 1 vs 2 $p = 0.008$
